# IL-6 Inhibition Reduces STAT3 Activation and Enhances the Antitumor Effect of Carboplatin

**DOI:** 10.1155/2016/8026494

**Published:** 2016-03-02

**Authors:** Zhi-Yong Wang, Jun-Ai Zhang, Xian-Jin Wu, Yan-Fang Liang, Yuan-Bin Lu, Yu-Chi Gao, You-Chao Dai, Shi-Yan Yu, Yan Jia, Xiao-Xia Fu, Xiaoquan Rao, Jun-Fa Xu, Jixin Zhong

**Affiliations:** ^1^Guangdong Provincial Key Laboratory of Medical Molecular Diagnostics, No. 1 Xincheng Road, Dongguan 523808, China; ^2^Department of Clinical Immunology, Institute of Laboratory Medicine, Guangdong Medical University, No. 1 Xincheng Road, Dongguan 523808, China; ^3^Department of Laboratory Medicine, Affiliated Hospital of Guangdong Medical University, Zhanjiang 524001, China; ^4^Department of Pathology, Dongguan 5th Hospital, Dongguan 523905, China; ^5^Department of Medicine, University of Maryland School of Medicine, Baltimore, MD 21201, USA

## Abstract

Recent studies suggest that tumor-associated macrophage-produced IL-6 is an important mediator within the tumor microenvironment that promotes tumor growth. The activation of IL-6/STAT3 axis has been associated with chemoresistance and poor prognosis of a variety of cancers including colorectal carcinoma and thus serves as a potential immunotherapeutic target for cancer treatment. However, it is not fully understood whether anticytokine therapy could reverse chemosensitivity and enhance the suppressive effect of chemotherapy on tumor growth. In this study, we aimed to investigate the effect of IL-6 inhibition therapy on the antitumor effect of carboplatin. Enhanced expression of IL-6 and activation of STAT3 were observed in human colorectal carcinoma samples compared to normal colorectal tissue, with higher levels of IL-6/STAT3 in low grade carcinomas. Treatment of carboplatin (CBP) dose-dependently increased IL-6 production and STAT3 activation in human colorectal LoVo cells. Blockade of IL-6 with neutralizing antibody enhanced chemosensitivity of LoVo cells to carboplatin as evidenced by increased cell apoptosis. IL-6 blockade abolished carboplatin-induced STAT3 activation. IL-6 blockade and carboplatin synergistically reduced cyclin D1 expression and enhanced caspase-3 activity in LoVo cells. Our results suggest that inhibition of IL-6 may enhance chemosensitivity of colon cancers with overactive STAT3 to platinum agents.

## 1. Introduction

IL-6, produced by tumor-associated macrophage, is an important mediator that promotes tumor growth [1, 2]. Although there was evidence supporting a role in T-cell activation and trafficking [3], IL-6 within the tumor microenvironment is generally considered as a malevolent player that promotes tumor progression. By activating downstream Janus kinase (JAK) signal transducer and activator of transcription-3 (STAT3) signaling, IL-6 promotes cancer cell proliferation, survival, and metastatic dissemination. In addition, IL-6 may also act on other cell types within the tumor microenvironment to enhance tumor growth by supporting angiogenesis [4] and immune escape [5, 6].

Platinum drugs such as cisplatin, carboplatin, and oxaliplatin are a class of chemotherapy agents that trigger apoptosis of tumor cells by binding to and causing DNA cross-linking. They are widely used in cancer chemotherapy due to their broad spectrum of activities against several solid tumors [7]. However, the drug resistance is a major problem in platinum-based therapy, with 75% relapse for cisplatin [8]. Enhanced activation of STAT3 has been suggested as a major contributor to platinum resistance [9, 10]. In this investigation, we examine the effect of carboplatin (CBP) and IL-6 blockade combination therapy on the growth of LoVo, a human colon carcinoma cell line.

## 2. Materials and Methods

### 2.1. Human Colorectal Carcinoma Tissue Collection

Colorectal tumor and nontumor colon tissue samples were collected at the time of surgical resection at Dongguan 6th Hospital. All procedures involving human participants were approved by the Research Ethics Board and the Institutional Review Board (IRB) at the Guangdong Medical College and Dongguan 6th Hospital. Written informed consent was obtained before tissue collection.

### 2.2. Cell Culture and Reagents

The human colorectal cancer LoVo cells were purchased from ATCC (Manassas, VA, USA). LoVo cells were cultured in F12K medium supplemented with 10% fetal bovine serum, 100 g/mL streptomycin, and 100 U/mL penicillin, at 37°C, 5% CO_2_, and high humidity. The sources of antibodies (Abs) were as follows: IL-6 was purchased from R&D (Minneapolis, MN, USA), p-STAT3 was purchased from Abcam (Cambridge, MA, USA), cleaved caspase-3 was purchased from Cell Signaling (Beverly, MA, USA), and STAT3, cyclin D1, GAPDH, and the HRP-labeled secondary antibodies were purchased from EnoGene (Nanjing, China). Carboplatin was purchased from MelonePharma (Dalian, Liaoning, China). Annexin-V-FITC apoptosis detection kit, DAB Substrate Kit, and Cell Counting Kit-8 (CCK-8) were purchased from Beyotime (Beyotime, Shanghai, China). IL-6 ELISA kit was from NeoBioscience (Shenzhen, Guangdong, China).

### 2.3. Immunohistochemistry Detection

All human colorectal tumor and nontumor specimens were fixed in 10% neutral-buffered formalin, dehydrated in ascending series of ethanol, and routinely embedded in paraplast. Sections were cut at 10 *μ*m and overnight stained with indicated primary antibodies after deparaffinization, rehydration, antigen recovery, and blocking. After washing, sections were incubated with HRP-labeled corresponding secondary antibodies and the signal was developed with a DAB Substrate Kit (Beyotime, Shanghai, China).

### 2.4. ELISA Detection of IL-6

LoVo cells were treated with indicated concentration of CBP or vehicle for 48 h. Culture supernatant was collected for the detection of IL-6 using an ELISA kit (NeoBioscience) according to the manufacturer's instruction.

### 2.5. Apoptosis Detection

Cell apoptosis was measured by an Annexin-V-FITC apoptosis detection kit (Beyotime, Shanghai, China) following the manufacturer's instruction. Briefly, cells were incubated with 5 *μ*Mol/L Annexin-V and 1 *μ*g/mL propidium iodide (PI) at room temperature for 15 min. Cells were then analyzed on a BD FACSCalibur cytometer within 1 h.

### 2.6. Western Blot

Cells were lyzed after 24 hours of indicated treatment and subjected to western blot detection of p-STAT3, STAT3, cyclin D1, cleaved caspase-3, and GAPDH as described [11]. Briefly, the blots were probed with an indicated primary Ab followed by an HRP-conjugated secondary antibody. The reactive bands were visualized using an ECL western blot kit.

## 3. Results

### 3.1. IL-6 Expression and STAT3 Activation in Human Colorectal Carcinoma

**Figure 1:**
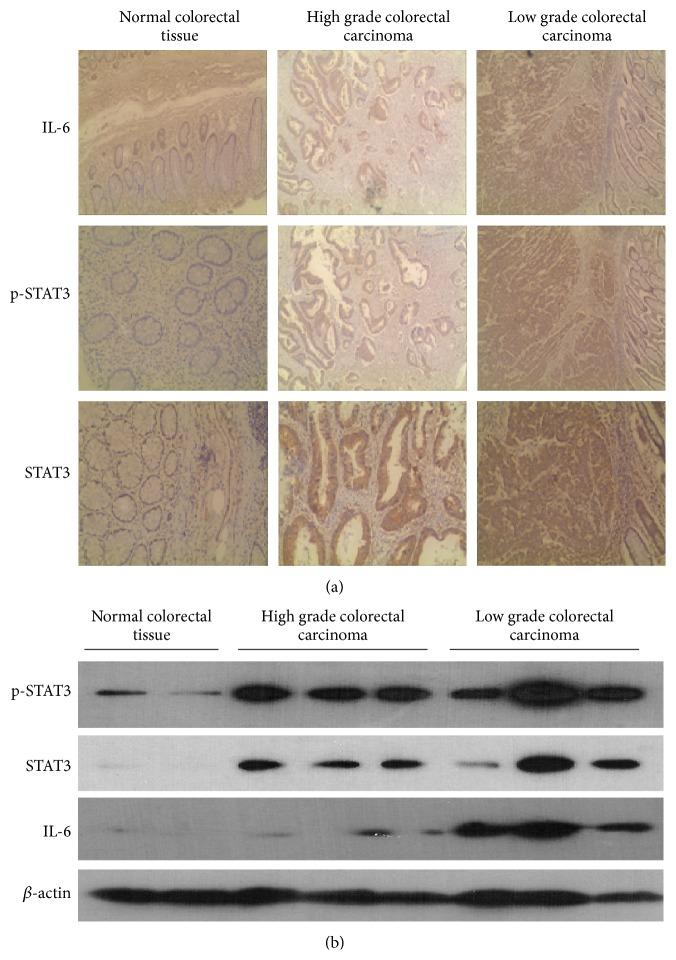
IL-6 expression and STAT3 activation in human colorectal carcinoma: (a) human colorectal carcinoma and nontumor samples were collected at surgery and paraffin-embedded sections were used for the immunohistochemistry detection of IL-6, p-STAT3, and STAT3. Representative images (200x magnification) were shown. (b) Western blot showing the expression of p-STAT3, STAT3, IL-6, and *β*-actin (internal control) in normal colorectal tissue, high grade colorectal carcinoma, and low grade colorectal carcinoma.

Increased expression of IL-6 has been detected and associated with an unfavorable prognosis in patients with various types of cancers including breast cancer, colorectal carcinoma, and ovarian cancer [12–17]. To confirm whether IL-6-STAT3 axis is activated in colon cancer, human colorectal carcinoma and matched nontumor colon tissue samples were used for the immunohistochemistry detection of IL-6, phosphor-STAT3 (p-STAT3), and STAT3. As shown in Figure 1, IL-6 expression increased in colorectal carcinoma especially in low grade carcinoma. Consistent with this, both the activation of STAT3 and expression of total STAT3 were upregulated in colorectal carcinoma and higher levels were seen in low grade carcinoma (Figure 1(a)). These results were further confirmed by western blot detection. Levels of IL-6, STAT3, and p-STAT3 were all increased in the human colorectal carcinoma samples, with a higher increase in low grade carcinoma (Figure 1(b)).

### 3.2. Effect of Carboplatin (CBP) Treatment on IL-6 Production by LoVo Cells

**Figure 2:**
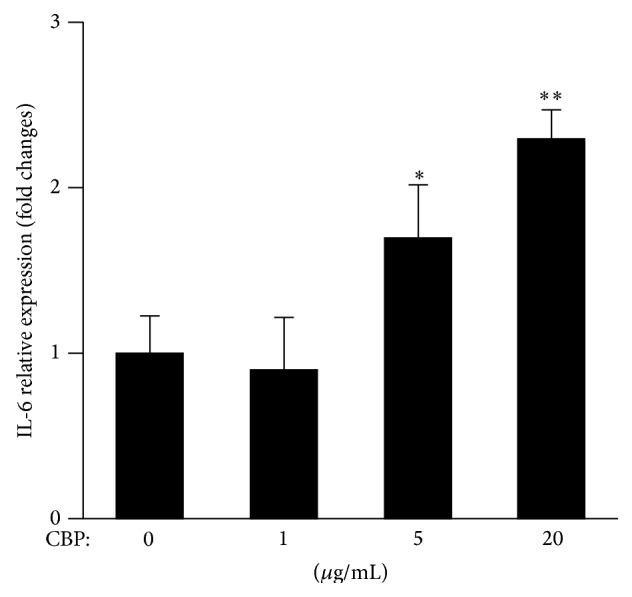
CBP treatment increased IL-6 production by LoVo cells: LoVo cells were treated with CBP at indicated doses for 48 h. Culture supernatant was collected for ELISA detection of IL-6. ^*∗*^ *P* < 0.05; ^*∗∗*^ *P* < 0.01.

Enhanced activation of STAT3 has been suggested to associate with the chemoresistance of platinum agents [9, 10]. To examine whether CBP treatment could affect IL-6 production in tumor, LoVo cells were treated with different concentrations of CBP for 48 h. Treatment with 1 *μ*g/mL CBP did not affect IL-6 level in the culture supernatant, while both 5 and 20 *μ*g/mL CBP treatments increased IL-6 production with a dose-dependent effect (Figure 2). Treatment of 20 *μ*g/mL CBP resulted in an over 2-fold increase in IL-6 secretion. Since IL-6 has been suggested to promote tumor survival [1, 2], CBP-induced IL-6 production might contribute to drug resistance to platinum.

### 3.3. Synergistic Effect of CBP and IL-6 Blockade on Colorectal Cancer Cell Apoptosis

**Figure 3:**
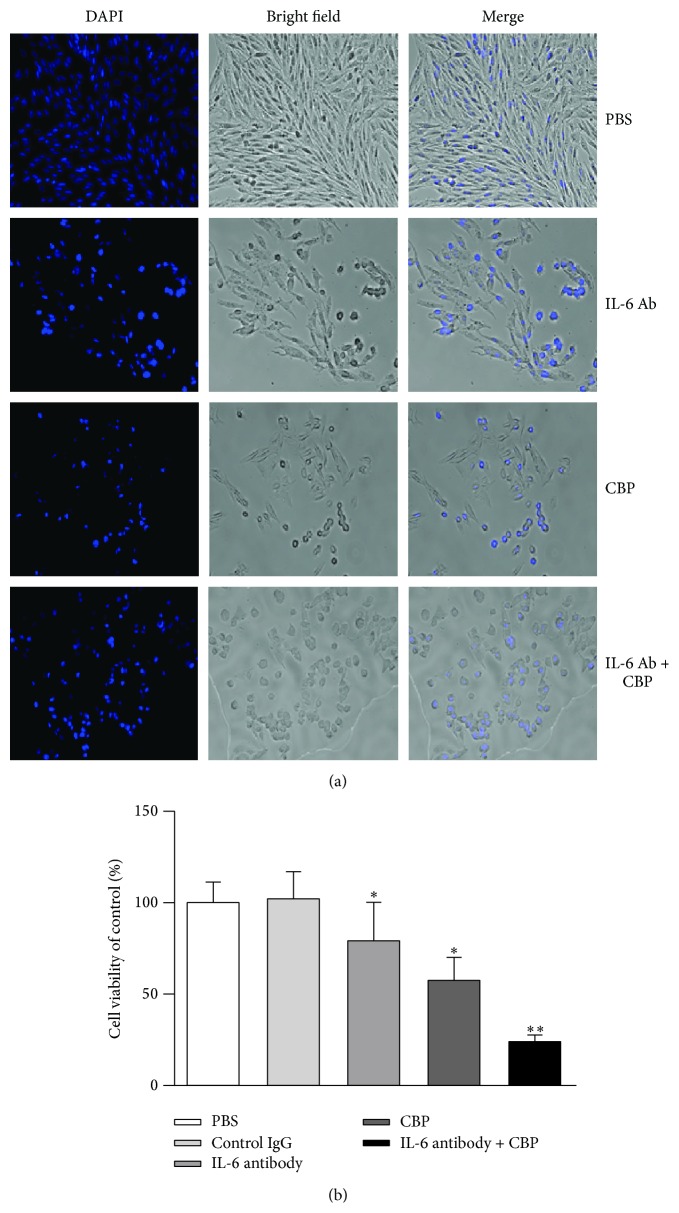
Synergistic effect of CBP and IL-6 blockade on LoVo cell survival: (a) LoVo cells were treated with 20 *μ*g/mL CBP and/or 500 *μ*g/mL IL-6 neutralizing antibody (Ab) or PBS. Cells were stained with DAPI (0.2 *μ*g/mL) and morphological change was examined after 72 h under microscope (100x). (b) LoVo cells were treated with 20 *μ*g/mL CBP and/or 500 *μ*g/mL IL-6 neutralizing antibody (Ab) or PBS. Cells were collected for the detection of viability using CCK-8 kit. ^*∗*^ *P* < 0.05; ^*∗∗*^ *P* < 0.01.

Increased production of IL-6 and enhanced activation of STAT3 have been suggested to associate with platinum resistance [9, 10, 18]. To test the effect of IL-6 blockade on CBP chemosensitivity, cell viability and apoptosis of LoVo cells were examined 72 hours after treatment of IL-6 neutralizing antibody (Ab) and/or CBP. As shown in Figure 3(a), a large amount of CBP-treated or IL-6-Ab-treated cells changed their shape from a flat adherent to a rounded morphology, indicating an early stage of apoptosis. Combined treatment of IL-6 blockade and CBP significantly enhanced this change with most of the cells showing apoptotic morphological changes. Consistent with that, cell viability assay by CCK-8 confirmed that CBP treatment induced cell death/CCK-8 release and IL-6 blockade further promoted cell death in CBP-treated LoVo cells (Figure 3(b)).

**Figure 4:**
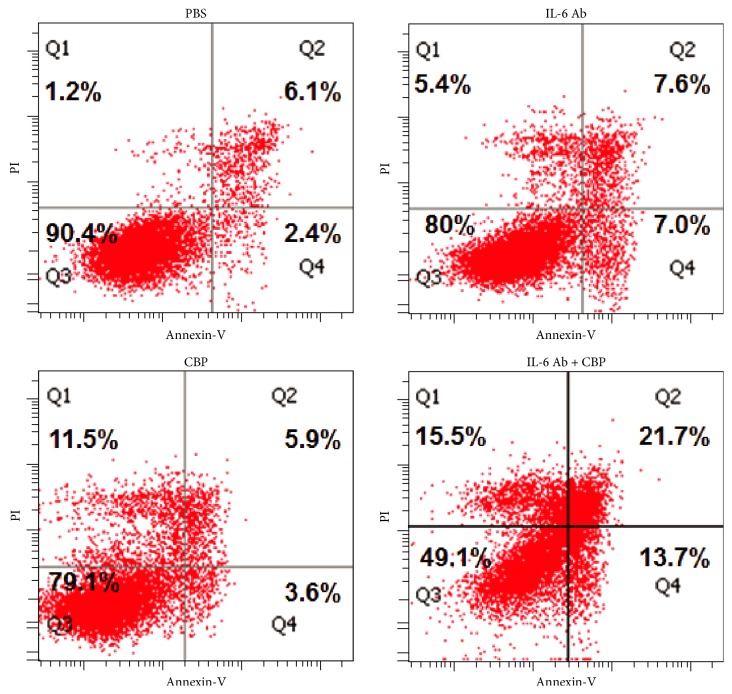
Effect of CBP and IL-6 blockade on LoVo cell apoptosis: LoVo cells were treated with 20 *μ*g/mL CBP, 500 *μ*g/mL IL-6 neutralizing antibody (Ab), or both combined or PBS for 24 h. After staining with Annexin-V-FITC and PI, cells were analyzed on a flow cytometer.

To further confirm the apoptosis induced by CBP and IL-6 blockade, LoVo cells after indicated treatments were stained with Annexin-V and PI and analyzed on a flow cytometer. Results showed that both IL-6 Ab and CBP treatment increased apoptotic and necrotic cell number. Although frequencies of dead cells (cells in quadrants Q1, Q2, and Q4) were at a similar level in IL-6 Ab- and CBP-treated groups, the CBP-treated cells showed higher number of necrotic cells (quadrant Q1: 1.2% versus 5.4% versus 11.5% for cells treated with PBS versus IL-6 Ab versus CBP, resp.) while IL-6-treated cells displayed higher number of apoptotic cells (quadrant Q4: 2.4% versus 7.0% versus 3.6% for cells treated with PBS versus IL-6 Ab versus CBP, resp.). Combined treatment of IL-6 Ab + CBP dramatically enhanced both apoptosis (1.2% versus 15.5% for PBS versus IL-6 Ab + CBP, Figure 4) and necrosis (2.4% versus 13.7% for PBS versus IL-6 Ab + CBP, Figure 4).

### 3.4. Effect of IL-6 Blockade and CBP on Pro- and Antisurvival Molecules

**Figure 5:**
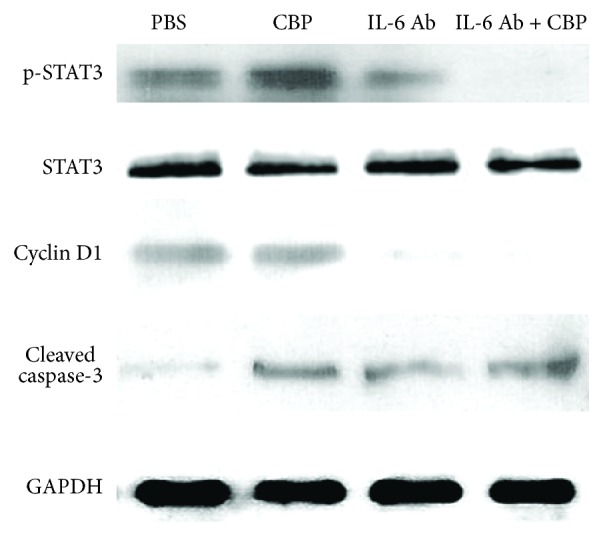
Western blot analysis of STAT3 and downstream molecules: LoVo cells were treated with 20 *μ*g/mL CBP, 500 *μ*g/mL IL-6 neutralizing antibody (Ab), or both combined or PBS control for 24 h. Total proteins were then isolated for the western blot detection of p-STAT3, total STAT3, cyclin D1, cleaved caspase-3, and GAPDH.

Western blot was then used to confirm the effect of CBP and IL-6 blockade on STAT3 activation. Consistent with the increase of IL-6 after CBP treatment, CBP enhanced the activation of STAT3 as evidenced by increased p-STAT3 level, while IL-6 blockade suppressed STAT3 activation (Figure 5). Cyclin D1, a well-described downstream target for STAT3 [19], was also increased by CBP and reduced by IL-6 neutralization (Figure 5). Cleaved caspase-3 is an apoptosis marker and is indispensable for cell apoptosis [20]. Both CBP and IL-6 blockade increased the activation of caspase-3, while combined treatment of IL-6 blockade and CBP further increased the activation of caspase-3 (Figure 5).

## 4. Discussion

Combination therapy is the future direction for cancer treatment. More and more clinical evidence showed that monotherapy such as surgery, chemotherapy, or radiation therapy alone does not provide a satisfactory result for cancers. Chemotherapy is one of the most commonly used traditional therapies for cancer. However, studies indicate that most chemotherapy agents are detrimental to immunity. Therefore, immunotherapy or biologic therapy is increasingly used in combination with chemotherapy, a strategy referred to as “biochemotherapy” or “chemoimmunotherapy” [21–25]. Of all the currently employed combination therapeutic strategies, platin-based regimen combined with biologic agents has attained the highest attention [26]. In the current study, we investigate the effect of anti-IL-6 therapy on CBP therapy.

IL-6 belongs to a cytokine family signaling through a common receptor gp130. The binding of IL-6 to its receptor results in the activation of the associated Janus kinases (JAKs), followed by the recruitment and activation of STAT3 and STAT1 [27]. Although an in vitro study suggests that IL-6 suppresses in vitro growth of some cancer cells [28], IL-6/STAT3 has been shown to promote tumor progression and immune escape in a variety of in vivo models [4–6]. Deficiency in STAT3 has been shown to protect against colitis-associated colorectal cancer in mice [29]. Therefore, IL-6/STAT3 has been suggested as a potential immunotherapeutic target for malignant diseases [6, 30]. By using immunohistochemistry, western blot, and ELISA, we found in this study that IL-6 is upregulated in colorectal carcinoma especially in low grade carcinoma, accompanied with enhanced STAT3 activation. In addition to active STAT3 (p-STAT3), total STAT3 protein level was also increased in both high grade and low grade colorectal carcinomas. This is probably because long term activation of STAT3 by IL-6 enhances the transcription and expression of STAT3. This result is consistent with the findings by other groups that IL-6 increased during tumorigenesis [31, 32].

Carboplatin, also known as cis-diammine(1,1-cyclobutanedicarboxylato)platinum(II), is used as an anticancer chemotherapy drug for a variety of cancer types due to its broad spectrum of activities against several solid tumors [7]. It triggers tumor cell apoptosis by causing DNA cross-linking. However, its use in clinic is largely limited by the high incidence of drug resistance. Studies suggested that the activation of STAT3 is enhanced in platinum therapy and is responsible for platinum resistance [9, 10]. We therefore hypothesize that IL-6 inhibition could improve platinum resistance. We first confirmed that CBP dose-dependently increased IL-6 production by LoVo cells. IL-6 blockade had a synergistic effect on promoting tumor cell apoptosis when combined with CBP.

CBP has been shown to induce cancer cell apoptosis via activation of caspases [33]. We verified this effect by detecting the viability and apoptosis rate. We also examined the effect of IL-6 blockade on tumor cell apoptosis with or without the presence of CBP. Our results suggest that IL-6 blockade alone reduces tumor cell viability and promotes apoptosis. Furthermore, IL-6 blockade and CBP have synergistic effect on promoting apoptosis.

As a transcription factor, STAT3 mediates the expression of various genes including Bcl-XL, survivin, and cyclin D1 [34, 35]. In our study, we confirmed that both IL-6 blockade and CBP activate caspase-3. Combined treatment of IL-6 blockade and CBP had a synergistic effect on caspase-3 activation. CBP therapy enhanced STAT3 activation while IL-6 blockade eliminated STAT3 activation in both CBP-treated LoVo cells and vehicle control. In consistency with that, cyclin D1 slightly increased in CBP-treated cells, while IL-6 blockade dramatically diminished cyclin D1 expression in both CBP-treated LoVo cells and vehicle control. These suggest that IL-6 blockade may block the adverse effect of CBP-induced IL-6 upregulation and it has a synergistic effect on enhancing proapoptotic signaling.

In summary, our results indicate that IL-6 blockade may enhance the antitumor effect of CBP and eliminate the adverse effects caused by CBP-induced IL-6 upregulation. However, further studies are required to confirm this effect in vivo.
